# Assessment of variation in 30‐day mortality following cancer surgeries among older adults across US hospitals

**DOI:** 10.1002/cam4.2800

**Published:** 2020-01-09

**Authors:** Allison Lipitz‐Snyderman, Jessica A. Lavery, Peter B. Bach, Diane G. Li, Annie Yang, Vivian E. Strong, Ashley Russo, Katherine S. Panageas

**Affiliations:** ^1^ Center for Health Policy and Outcomes Department of Epidemiology and Biostatistics Memorial Sloan Kettering Cancer Center New York NY USA; ^2^ Biostatistics Service Department of Epidemiology and Biostatistics Memorial Sloan Kettering Cancer Center New York NY USA; ^3^ Department of Surgery Memorial Sloan Kettering Cancer Center New York NY USA; ^4^ Present address: GoodRx Santa Monica CA USA; ^5^ Present address: Rutgers New Jersey Medical School Rutgers, The State University of New Jersey Newark NJ USA; ^6^ Present address: Department of Surgery University of Massachusetts Medical Center Worcester MA USA

**Keywords:** complications, health care, outcomes assessment (health care), quality indicators, quality of health care, surgery

## Abstract

**Background:**

While public reporting of surgical outcomes for noncancer conditions is common, cancer surgeries have generally been excluded. This is true despite numerous studies showing outcomes to differ between hospitals based on their characteristics. Our objective was to assess whether three prerequisites for quality assessment and reporting are present for 30‐day mortality after cancer surgery: low burden for timely reporting, hospital variation, and potential for public health gains.

**Study Design:**

We used Fee‐for‐Service (FFS) Medicare claims to examine the extent of variation in 30‐day cancer surgical mortality between 3860 US hospitals. We included 340 489 surgeries for 12 cancer types for FFS Medicare beneficiaries aged ≥66 years, 2011‐2013. Hierarchical mixed‐effects logistic regression models adjusted for patient and hospital characteristics and with a random hospital effect were fit to obtain hospital‐specific risk‐standardized mortality rates (RSMRs) and 99% confidence intervals (CI). We calculated a hospital odds ratio to describe the difference in mortality risk for a hospital above vs below average quality and estimated the potential mortality reduction.

**Results:**

The median number of cancer surgeries per hospital was 34. The median RSMR overall was 2.41% (99% CI 2.28%, 2.66%). In aggregate and for most cancers, variation between hospitals exceeded that due to differences in patient and hospital characteristics. For individual cancers, relative differences exceeded 20% in mortality risk between patients undergoing surgery at a hospital below vs above average quality, with the potential for an estimated 500 deaths prevented annually given hypothetical improvements.

**Conclusion:**

Quality measurement and reporting of 30‐day mortality for cancer surgery is worthy of consideration.

## Graphical abstract

Given the potential opportunity to improve outcomes, this study sought to assess 30‐day mortality after cancer surgery across U.S. hospitals. Using national Fee‐for‐Service Medicare claims, there was significant variation between 3860 hospitals, with as many as 500 avoidable deaths annually.

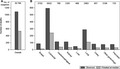

## 1 INTRODUCTION

While decades of research have raised concerns about the inconsistent quality of cancer surgery in the United States,^1^, ^2^, ^3^, ^4^, ^5^, ^6^, ^7^, ^8^ measures of surgical outcomes, so prevalent for other conditions, have not been included specifically for cancer surgery in national public reporting efforts. Policymakers and payers have embraced quality measurement and reporting as potent means by which to improve patient outcomes, both through feedback and payment incentives that can spur institutional quality improvement efforts, and by patients using reported measures when they choose where to receive their care.^9^, ^10^, ^11^, ^12^

Numerous studies have suggested that outcomes are variable between hospitals. Strong correlations have been documented for both short‐ and long‐term outcomes following specific cancer surgeries in relation to hospital factors such as surgical volume, teaching status, and geographic region.^1^, ^2^, ^3^, ^4^, ^5^, ^6^, ^7^, ^8^, ^13^ Surgery is a primary modality of cancer treatment: over 87% of patients with breast and colorectal cancers, 52% with lung cancer, and 24% with prostate cancer undergo surgery.^14^

Despite consistent evidence of outcome variation by hospital characteristics, the Centers for Medicare & Medicaid (CMS) Hospital Compare website, a national program that publicly reports hospital‐specific measures of quality,^15^ does not include surgical outcome measures for cancer. The CMS prospective payment system (PPS) exempt cancer hospitals reporting program also does not include outcomes from cancer surgery. By fiscal year 2021 the only planned surgical outcome measure for the PPS‐exempt hospital reporting program is procedure‐specific surgical site infection.^16^, ^17^ Even if included for PPS‐exempt cancer hospitals, there are only 11 of these facilities, and studies generally suggest their outcomes are above average, so measuring their performance only has limited potential to improve cancer outcomes.^17^, ^18^ Other programs such as the American College of Surgeon's National Surgical Quality Improvement Program collect surgical outcomes data for quality improvement, but hospital‐level public reporting is limited.^19^, ^20^, ^21^

At least three conditions should be met if comparative hospital performance measurement and reporting of cancer surgical outcomes is to be pursued: the outcome can be captured efficiently and rapidly; there are important variations in the outcome at baseline; and the number of people potentially affected if the outcome were to improve is sizable.^20^, ^22^, ^23^ We hypothesized that cancer surgery would be a promising area for measurement, providing the motivation to examine these three conditions using the foundational measure of 30‐day mortality. We examined these conditions using a national source, the Fee‐for‐Service (FFS) Medicare claims dataset, with 3,860 US hospitals performing cancer surgery between 2011 and 2013 for 12 cancer types.

## 2 MATERIALS AND METHODS

### 2.1 Data source and cohort

The national FFS Medicare 100% Research Identifiable Files were used for this analysis. These include inpatient, outpatient, carrier, durable medical equipment, hospice, home health, skilled nursing facility, Part D claims, vital status, and master beneficiary summary files. Using previously published methods, beneficiaries aged 66 years or older undergoing a cancer surgery in 2011‐2013 were identified. The analysis was limited to cancers where ≥80% of procedures in Medicare claims alone matched those in the gold standard SEER‐Medicare dataset for condition and procedure, and for which false identification of a cancer‐directed surgery occurred at a frequency less than 3%.^24^ Twelve categories of cancer surgeries qualified: bones and joints; breast; colorectal; gastroesophageal; kidney; liver; lung; other gynecologic; ovary; pancreas; prostate; and sarcoma. Inpatient surgeries were identified using ICD‐9‐CM procedure codes and HCPCS codes were additionally used to identify outpatient surgeries for breast cancer. If a patient had multiple cancer surgeries, the first for a given cancer site was included, and only surgeries for a second cancer site occurring more than 30 days following the prior surgery were included. The analysis was limited to patients with at least 12 months of continuous enrollment in Parts A and B of FFS Medicare preadmission required for comorbidity assessment, and 1 month of coverage postdischarge date or through death if the patient died within 30 days. Those discharged against medical advice or discharged after November 30, 2013 were excluded.

Surgeries were attributed to their hospital by their CMS Certification Number (CCN), as recorded at the time of surgery. We considered each CCN as a unique hospital, following CMS's reporting approach,^25^, ^26^ recognizing that hospital ownership and mergers might have occurred over the study period.^27^ Hospital characteristics were obtained from the American Hospital Association (AHA) survey (2012).^28^

### 2.2 Statistical analyses

We assessed hospitals’ cancer surgical volume, evaluated the extent of hospital variability in risk‐adjusted 30‐day all‐cause mortality after cancer surgery, and estimated the number of lives saved were poor‐performing hospitals to improve their quality. All analyses were performed by cancer site and in aggregate. A two‐sided *P* < .01 was considered the threshold for significance.

We conducted four sets of analyses. To test whether there was underlying variability, our primary analysis adjusted for patient characteristics that could predict 30‐day mortality. A second analysis also included hospital‐level characteristics to determine if variation was explained by hospital descriptors that are already available. We also ran these two models focused only on surgeries that were nonemergent. Patient characteristics adjusted for included age, sex, race, and Charlson comorbidity score (0, 1, ≥2)^29^, ^30^ in the year prior to surgery, and cancer site (for aggregate analyses). Although FFS Medicare claims do not include clinical information regarding cancer stage, we previously demonstrated that risk adjustment was not sensitive to the inclusion or absence of this information.^31^ Hospital characteristics came from the AHA database including hospitals’ location (rural/urban), organizational control (not‐for‐profit, private, government), and teaching status (defined as a member of the council of teaching hospitals of the American Medical Association).^28^ These characteristics were not available for 84 hospitals (2.2% of the sample) and were excluded from analyses that depended on these characteristics. Hospital volume was calculated as the total number of inpatient and outpatient surgeries performed over the 3‐year study, dichotomized at the 75th percentile.

Hierarchical mixed‐effects logistic regression models with a random effect for hospital were fit to obtain adjusted mortality rates. This approach accommodates the hierarchical structure of the data accounting for the correlation of outcomes among patients from the same hospital*.* 32 Hospital‐specific risk‐standardized mortality rates (RSMRs) were calculated as the predicted value, which was derived from the random effects model, divided by the expected value, which was derived from a logistic regression model without a random hospital effect. This ratio was then multiplied by the national 30‐day mortality rate ($\bar{y}$) to obtain a relative measure of performance: a RSMR greater than $\bar{y}$ indicates that the performance at a given hospital was poorer than expected, whereas one lower than $\bar{y}$ indicates that the performance at a given hospital was better than expected. Utilizing this approach, low volume hospitals that have empirically poor performance will have a RSMR closer to the mean than a larger volume hospital with equally poor empiric performance. As our intent was to determine the extent of variation, the use of this approach was conservative.

We assessed between‐hospital variation in 30‐day mortality by examining the distribution of RSMR's at the hospital level. We tested for variation using a Wald test of the random effect. A two‐sided *P* < .01 was considered the threshold for significance for a conclusion that there was underlying variation. We further quantified the variation between hospitals by computing hospital odds ratios (hORs) and 99% confidence intervals based on the standard deviation (SD) of the random effect. With a single covariate *X* and a hospital random effect ω, where *i* indexes patients and *j* indexes hospitals, the log‐odds of 30‐day mortality are modeled by:

$$\text{logit}\left(\rho_{\mathrm{ij}}\right)=\beta_{0}+\beta_{1}X_{\mathrm{ij}}+\omega_{j}$$

To compare the risk of mortality for a patient treated at a hospital whose mortality is 1 SD above average (ie, $\omega_{1}=+1\times \text{SD}$) to a patient with the same covariates treated at a hospital whose mortality is 1 SD below average (ie, $\omega_{2}=-1\times \text{SD}$), the OR comparing mortality between these two patients is *e* ^2SD^. The hOR represents the odds of 30‐day mortality given that a patient underwent surgery at a hospital below average (+1 SD) quality vs above average quality (−1 SD).^4^

Based on the distribution of RSMRs, if hospitals performing in the upper quartile were to improve performance to the median, the reduction in mortality represents the estimated number of lives saved. We calculated the estimated number of lives saved from the models of nonemergent surgeries overall and by cancer site.

### 2.3 Approvals

Centers for Medicare & Medicaid approved the use of the FFS Medicare files for this analysis, which was deemed exempt research by the Memorial Sloan Kettering Cancer Center Institutional Review Board. Analyses were performed in SAS (Version 9.4, Cary, NC).

## 3 RESULTS

Across all cancer sites, there were 340 489 surgeries performed for FFS Medicare beneficiaries at 3860 US hospitals. Most patients were female (66.8%), white (88.5%), and had one or more comorbidities (55.3%). For breast cancer, 78.9% of surgeries were outpatient. Emergent surgeries accounted for between 0.4% (prostate) and 20.6% (colorectal) of surgeries. The 30‐day mortality rate overall was 2.4%; for breast and prostate cancer, it was less than 1%. The mortality rate was over 5% for colorectal and gastroesophageal cancer surgeries (Table 1).

**Table 1:** Characteristics of fee‐for‐service Medicare beneficiary cancer surgeries, 2011‐2013

	Overall	Bones and joints	Breast	Colorectal	Gastroesophageal	Kidney	Liver	Lung	Other gynecologic	Ovary	Pancreas	Prostate	Sarcoma
Number of cancer surgeries^a^	340 489	873	119 217	85 857	7899	24 578	3562	33 513	18 603	8805	6391	29 207	1984
30‐day mortality rate	2.4	2.1	0.2	5.1	5.9	1.6	4.3	4.8	1.4	3.5	4.3	0.2	1.3
Year of surgery^b^													
2011	34.6	32.4	33.1	35.5	32.6	33.9	28.9	33.8	37.0	34.2	32.2	40.3	31.7
2012	34.6	34.1	35.0	34.5	35.7	34.8	36.5	34.9	34.4	34.5	35.8	31.6	34.7
2013	30.8	33.4	31.9	30.0	31.7	31.3	34.6	31.4	28.6	31.3	32.0	28.1	33.6
Age (y)													
66‐69	22.1	18.3	20.5	13.8	19.5	23.9	23.3	21.2	25.4	23.6	21.1	51.1	15.9
70‐74	28.2	27.4	27.4	22.0	30.1	31.8	34.2	32.5	29.4	30.5	30.8	39.2	24.0
75‐79	21.8	19.7	22.4	22.3	24.6	23.4	25.6	26.6	21.1	23.5	26.7	8.7	22.4
80‐84	15.8	20.7	16.6	21.0	15.9	14.3	12.6	14.9	14.3	14.8	15.9	0.8	19.5
85+	12.0	13.9	13.1	20.8	9.9	6.5	4.3	4.9	9.9	7.6	5.5	0.1	18.2
Sex													
Female	66.8	47.2	98.9	54.1	34.6	40.5	47.4	49.7	100	100	50.6	‐	48.4
Male	33.2	52.8	1.1	45.9	65.4	59.5	52.6	50.3	‐	‐	49.4	100	51.6
Race													
White	88.5	90.5	89.2	88.0	80.1	87.9	84.1	91.2	86.4	90.7	88.2	88.1	89.2
Nonwhite	11.5	9.5	10.8	12.0	19.9	12.1	15.9	8.8	13.6	9.3	11.8	11.9	10.8
Charlson comorbidity score													
0	44.7	43.8	51.7	39.4	32.5	37.0	36.0	23.4	48.8	54.7	35.4	63.8	45.0
1	26.7	23.9	25.8	26.2	28.5	25.7	27.8	33.3	26.5	25.0	30.4	24.0	26.2
2+	28.6	32.3	22.5	34.5	38.9	37.4	36.2	43.3	24.7	20.3	34.2	12.2	28.8
Admission type^c^													
Nonemergent	93.1	96.0	99.4	79.4	89.8	97.1	96.1	96.0	96.4	92.4	94.0	99.6	94.7
Emergent	6.9	4.0	0.6	20.6	10.2	2.9	3.9	4.0	3.6	7.6	6.0	0.4	5.3

Note

N surgeries (column %) is shown.

a Patients could be included multiple times if they had surgeries for different cancer types.

b 2013 has fewer surgeries due to the inclusion criteria requiring 30 d of follow‐up.

c The flag for emergent admissions is only available on inpatient claims; it was assumed that outpatient surgeries (breast cancer) were nonemergent.

Among the 3776 (97.8%) hospitals with available hospital characteristics, the median number of cancer surgeries overall was 34 [interquartile range (IQR): 9, 108]. For hospitals performing at least one surgery, the median number was fewer than 10 for each cancer site, except for breast (median 17) and colorectal (median 14). Most surgeries were performed at not‐for‐profit hospitals and in urban locations. Bones and joints, liver, and sarcoma had the highest proportions of surgeries performed at teaching hospitals (>30%; Table 2). When including hospitals with nonemergent surgeries only, 23 (0.6%) hospitals were excluded (between 0.3% and 8.3% of hospitals excluded by site; Table A1).

**Table 2:** Characteristics of hospitals performing cancer surgeries in FFS Medicare 2011‐2013

	Overall	Bones and joints	Breast	Colorectal	Gastroesophageal	Kidney	Liver	Lung	Other gyn	Ovary	Pancreas	Prostate	Sarcoma
Total no. of hospitals performing surgery^a^	3776	302	3537	3471	1512	1998	556	1844	1615	1137	785	1600	506
No. of surgeries per hospital													
Mean	90	3	34	25	5	12	6	18	11	8	8	18	4
SD	150.2	3.2	47.1	29.2	9.4	18.0	10.4	27.7	19.0	11.3	14.8	33.5	7.0
Median^b^	34	<3	17	14	<3	6	3	9	3	3	3	7	<3
IQR^b^	9, 108	<3, 3	5, 44	5, 34	<3, 5	<3, 15	<3, 7	3, 23	<3, 12	<3, 10	<3, 8	<3, 21	<3, 4
Hospital location													
Rural (%)	35.6	5.3	35.2	34.4	12.4	17.0	5.6	14.0	18.8	12.8	5.2	15.9	10.1
Urban (%)	64.4	94.7	64.8	65.6	87.6	83.0	94.4	86.0	81.2	87.2	94.8	84.1	89.9
Organizational control													
Not‐for‐profit (%)	62.7	73.8	64.1	64.6	70.3	70.1	77.5	70.4	71.3	72.8	75.5	72.4	75.1
Private (%)	19.2	10.3	18.2	18.6	17.9	18.4	9.7	19.1	15.0	15.2	13.4	16.2	12.8
Government (%)	18.1	15.9	17.7	16.8	11.8	11.6	12.8	10.5	13.7	12.0	11.1	11.4	12.1
Teaching status													
Nonteaching (%)	93.2	53.3	92.8	92.7	84.5	87.7	64.2	86.8	85.4	80.0	72.4	85.4	65.8
Teaching (%)	6.8	46.7	7.2	7.3	15.5	12.3	35.8	13.2	14.6	20.0	27.6	14.6	34.2

Note

The values that describe the interquartile range encompass hospitals with various volumes of services within those ranges. For instance, a 25th percentile value of 5 means that 25% of the hospitals performing at least one of these procedures have a total volume between 1 and 5.

The column % is shown and the denominator is hospitals that performed one or more surgeries.

Abbreviations: IQR, interquartile range; SD, standard deviation.

a Only includes hospitals that could be matched to the American Hospital Association Survey of Hospitals 2012 (included n = 3776; total considered n = 3860 hospitals).

b Values less than three are suppressed.

The median hospital RSMR (Table 3) across all cancer sites was 2.41% (99% CI 2.28%, 2.66%). Breast had the lowest RSMR (median 0.24%) and gastroesophageal the highest (median 5.72%). The median hospital RSMRs were robust to additional adjustments of hospital characteristics. Where estimable, the RSMRs were generally lower in the models that excluded emergent surgeries. Based on the Wald test of the random effect, there was statistically significant variation across hospitals for cancers in aggregate and for breast, colorectal, gastroesophageal, kidney, lung, ovary, and pancreas cancers. Models for bones and joints, prostate, and sarcoma were not estimable. Inclusion of hospital characteristics explained some variability, and all cancers in aggregate, as well as breast, colorectal, lung, and ovarian cancer maintained statistically significance. The results from the model adjusting for patient characteristics for all surgeries were comparable to the results from the model adjusting for patient characteristics for nonemergent surgeries only, except for kidney cancer (Table 3).

**Table 3:** Between‐hospital variation in 30‐day cancer surgical mortality and hospital odds ratio, by model

	Model including patient characteristics			Model including patient and hospital characteristics		
	Median RSMR (IQR)	Test of variation^a^	Hospital odds ratio (99% confidence interval)^b^	Median RSMR (IQR)	Test of variation	Hospital odds ratio (99% confidence interval)
All surgeries						
Overall^c^	2.41 (2.28, 2.66)	<0.001	1.44 (1.42, 1.45)	2.41 (2.31, 2.58)	<0.001	1.30 (1.29, 1.31)
Breast	0.24 (0.22, 0.24)	<0.001	4.31 (4.13, 4.52)	0.23 (0.22, 0.24)	<0.001	3.54 (3.41, 3.68)
Colorectal	5.06 (4.80, 5.60)	<0.001	1.39 (1.37, 1.40)	5.07 (4.85, 5.47)	<0.001	1.30 (1.29, 1.31)
Gastroesophageal	5.72 (5.48, 5.82)	0.002	1.50 (1.47, 1.53)	5.81 (5.69, 5.88)	0.080	1.21 (1.20, 1.22)
Kidney	1.54 (1.48, 1.58)	0.002	1.57 (1.54, 1.60)	1.55 (1.50, 1.58)	0.036	1.35 (1.34, 1.37)
Liver	4.20 (4.01, 4.27)	0.023	1.64 (1.59, 1.71)	4.19 (4.06, 4.26)	0.023	1.58 (1.53, 1.64)
Lung	4.73 (4.39, 5.56)	<0.001	1.55 (1.52, 1.58)	4.76 (4.54, 5.24)	<0.001	1.30 (1.29, 1.32)
Other gynecologic	1.40 (1.38, 1.41)	0.066	1.26 (1.25, 1.28)	1.41 (1.38, 1.41)	0.102	1.21 (1.20, 1.22)
Ovary	3.42 (3.27, 3.47)	0.004	1.55 (1.52, 1.59)	3.43 (3.28, 3.48)	0.009	1.45 (1.43, 1.49)
Pancreas	4.17 (3.89, 4.24)	0.001	1.89 (1.82, 1.98)	4.23 (4.05, 4.40)	0.030	1.37 (1.34, 1.39)
Nonemergent surgeries^d^						
Overall	1.73 (1.65, 1.91)	<0.001	1.43 (1.42, 1.45)	1.73 (1.66, 1.84)	<0.001	1.30 (1.29, 1.31)
Breast	Not estimable	Not estimable	Not estimable	0.20 (0.20, 0.21)	<0.001	4.69 (4.48, 4.93)
Colorectal	3.47 (3.28, 3.91)	<0.001	1.44 (1.43, 1.46)	3.49 (3.33, 3.79)	<0.001	1.31 (1.30, 1.32)
Gastroesophageal	4.88 (4.69, 4.97)	0.005	1.56 (1.53, 1.60)	4.96 (4.85, 5.02)	0.078	1.25 (1.24, 1.27)
Kidney	1.41 (1.36, 1.43)	0.036	1.40 (1.39, 1.42)	Not estimable	Not estimable	Not estimable
Liver	4.00 (3.81, 4.06)	0.022	1.68 (1.62, 1.75)	4.01 (3.89, 4.06)	0.022	1.61 (1.56, 1.68)
Lung	3.67 (3.45, 4.19)	<0.001	1.46 (1.44, 1.49)	3.71 (3.55, 3.99)	<0.001	1.28 (1.27, 1.29)
Other gynecologic	1.25 (1.23, 1.26)	0.040	1.34 (1.32, 1.36)	1.26 (1.23, 1.26)	0.063	1.28 (1.26, 1.29)
Ovary	2.93 (2.77, 2.98)	0.004	1.66 (1.62, 1.72)	2.93 (2.76, 2.98)	0.005	1.61 (1.57, 1.65)
Pancreas	3.94 (3.72, 3.99)	0.005	1.74 (1.68, 1.81)	3.97 (3.83, 4.01)	0.043	1.34 (1.32, 1.37)

Abbreviations: IQR, interquartile range; RSMR, risk‐standardized mortality rates.

a A test of whether the estimated variance of the random effects differed significantly from zero was used to evaluate hospital variation.

b The hospital odds ratio represents the odds of 30‐day mortality given that a patient underwent surgery at a hospital below average quality vs above average quality.

c Bones and joints, prostate, and sarcoma are included in aggregate analysis but results by cancer site are not estimable.

d The flag for emergent admissions is only available on inpatient claims; it was assumed that outpatient surgeries (breast cancer) were nonemergent.

The hOR for all cancer sites in aggregate was 1.44 (99% CI 1.42, 1.45), indicating that the odds of 30‐day mortality for a patient undergoing surgery at a hospital whose performance is below average are 44% higher than at a hospital whose performance is above average. Breast cancer had the largest hOR for all models; the odds of mortality were three‐ to four‐fold higher at a below average hospital as compared to an above average hospital, indicating that despite a low mortality rate (0.2%) and median hospital RSMR (0.24%) there are differences in the patients’ risk of 30‐day mortality between hospitals. Considering all models, pancreas had the second largest hospital odds ratio (hOR 1.89, 99% CI 1.82, 1.98, adjusted for patient characteristics) and gastroesophageal and other gynecologic cancers had the smallest (both hORs: 1.21, 99% CI 1.20, 1.22, adjusted for patient and hospital characteristics; Table 3).

The model adjusted for both patient and hospital characteristics (Table 4) showed that older patients, male patients, and those with a higher number of comorbidities had higher odds of mortality. Patients treated at hospitals with surgical volumes above the 75th percentile and teaching hospitals had lower odds of mortality. Patients treated at government hospitals had higher odds of mortality compared to those treated at not‐for‐profit hospitals. Patients’ odds of mortality did not differ by patient race or their hospitals’ location. For several of these characteristics, statistical significance of the output differed by cancer site.

**Table 4:** Adjusted odds ratios and 99% confidence intervals for the associations between patient and hospital chsaracteristics and 30‐day cancer surgery mortality

	Overall^a^	Breast	Colorectal	Gastroesophageal	Kidney	Liver
Age (y)						
66‐69	1 [Reference]	1 [Reference]	1 [Reference]	1 [Reference]	1 [Reference]	1 [Reference]
70‐74	1.18 (1.05, 1.32)*	1.13 (0.59, 2.14)	1.29 (1.06, 1.57)*	1.25 (0.81, 1.93)	1.22 (0.79, 1.86)	0.81 (0.41, 1.58)
75‐79	1.58 (1.42, 1.77)*	1.71 (0.93, 3.14)	1.75 (1.45, 2.12)*	1.47 (0.95, 2.27)	1.52 (0.99, 2.35)	1.41 (0.74, 2.68)
80‐84	2.18 (1.95, 2.44)*	2.68 (1.48, 4.86)*	2.58 (2.15, 3.10)*	1.76 (1.11, 2.78)*	2.14 (1.37, 3.36)*	1.62 (0.77, 3.42)
>85	3.97 (3.56, 4.44)*	4.79 (2.71, 8.49)*	4.65 (3.90, 5.55)*	3.22 (2.03, 5.11)*	3.29 (2.00, 5.41)*	3.26 (1.35, 7.86)*
Sex						
Female	1 [Reference]	1 [Reference]	1 [Reference]	1 [Reference]	1 [Reference]	1 [Reference]
Male	1.34 (1.26, 1.43)*	1.49 (0.45, 4.87)	1.28 (1.17, 1.39)*	1.18 (0.90, 1.54)	1.41 (1.07, 1.88)*	1.21 (0.77, 1.90)
Race						
Nonwhite	1 [Reference]	1 [Reference]	1 [Reference]	1 [Reference]	1 [Reference]	1 [Reference]
White	0.96 (0.87, 1.05)	0.56 (0.37, 0.86)*	1.02 (0.89, 1.16)	1.50 (1.06, 2.12)*	0.86 (0.58, 1.27)	0.91 (0.50, 1.66)
Charlson comorbidity score						
0	1 [Reference]	1 [Reference]	1 [Reference]	1 [Reference]	1 [Reference]	1 [Reference]
1	1.29 (1.19, 1.40)*	1.58 (1.04, 2.40)*	1.22 (1.09, 1.37)*	1.07 (0.75, 1.54)	1.35 (0.90, 2.02)	1.04 (0.57, 1.91)
>2	1.91 (1.78, 2.06)*	2.90 (2.00, 4.20)*	1.87 (1.69, 2.06)*	1.69 (1.24, 2.30)*	2.47 (1.76, 3.45)*	1.50 (0.88, 2.56)
Total FFS Medicare surgical volume						
<75th percentile	1 [Reference]	1 [Reference]	1 [Reference]	1 [Reference]	1 [Reference]	1 [Reference]
≥75th percentile	0.80 (0.73, 0.87)*	0.56 (0.36, 0.87)*	0.87 (0.78, 0.96)*	0.75 (0.55, 1.03)	0.78 (0.56, 1.07)	1.51 (0.78, 2.92)
Hospital location						
Rural	1 [Reference]	1 [Reference]	1 [Reference]	1 [Reference]	1 [Reference]	1 [Reference]
Urban	0.92 (0.83, 1.03)	0.85 (0.51, 1.42)	0.92 (0.81, 1.04)	0.89 (0.52, 1.52)	0.89 (0.54, 1.48)	1.07 (0.14, 7.99)
Organizational control						
Not‐for‐profit	1 [Reference]	1 [Reference]	1 [Reference]	1 [Reference]	1 [Reference]	1 [Reference]
Private	1.10 (0.98, 1.22)	0.95 (0.53, 1.68)	1.12 (0.98, 1.28)	1.12 (0.73, 1.72)	1.17 (0.77, 1.80)	4.38 (1.78, 10.78)*
Government	1.16 (1.04, 1.31)*	1.12 (0.62, 2.04)	1.15 (0.99, 1.34)	1.08 (0.71, 1.65)	1.31 (0.85, 2.02)	1.26 (0.59, 2.69)
Teaching status						
Nonteaching	1 [Reference]	1 [Reference]	1 [Reference]	1 [Reference]	1 [Reference]	1 [Reference]
Teaching	0.71 (0.64, 0.79)*	0.77 (0.39, 1.48)	0.71 (0.61, 0.83)*	0.67 (0.48, 0.93)*	0.75 (0.52, 1.08)	0.76 (0.41, 1.43)

	Lung	Other gynecologic	Ovary	Pancreas
Age (y)				
66‐69	1 [Reference]	1 [Reference]	1 [Reference]	1 [Reference]
70‐74	1.03 (0.84, 1.28)	1.12 (0.66, 1.90)	1.49 (0.87, 2.56)	1.06 (0.65, 1.72)
75‐79	1.50 (1.22, 1.85)*	1.56 (0.92, 2.66)	1.94 (1.13, 3.32)*	0.97 (0.59, 1.61)
80‐84	1.65 (1.31, 2.07)*	2.15 (1.25, 3.70)*	3.00 (1.74, 5.18)*	1.72 (1.03, 2.87)*
>85	3.20 (2.44, 4.19)*	3.21 (1.87, 5.51)*	4.68 (2.63, 8.35)*	1.70 (0.84, 3.41)*
Sex				
Female	1 [Reference]	—	—	1 [Reference]
Male	1.67 (1.45, 1.92)*	—	—	1.14 (0.82, 1.58)
Race				
Nonwhite	1 [Reference]	1 [Reference]	1 [Reference]	1 [Reference]
White	0.92 (0.73, 1.17)	0.63 (0.42, 0.96)*	0.96 (0.57, 1.61)	1.07 (0.65, 1.78)
Charlson comorbidity score				
0	1 [Reference]	1 [Reference]	1 [Reference]	1 [Reference]
1	1.36 (1.10, 1.69)*	1.45 (0.96, 2.19)	1.68 (1.15, 2.44)*	1.22 (0.80, 1.86)
>2	1.86 (1.53, 2.26)*	1.97 (1.34, 2.90)*	2.13 (1.47, 3.08)*	1.46 (0.98, 2.17)
Total FFS medicare surgical volume				
<75th percentile	1 [Reference]	1 [Reference]	1 [Reference]	1 [Reference]
≥75th percentile	0.65 (0.55, 0.76)*	0.78 (0.51, 1.19)	0.80 (0.55, 1.18)	0.50 (0.33, 0.77)*
Hospital location				
Rural	1 [Reference]	1 [Reference]	1 [Reference]	1 [Reference]
Urban	0.77 (0.59, 1.01)	1.86 (0.66, 5.23)	0.66 (0.32, 1.36)	0.82 (0.29, 2.31)
Organizational control				
Not‐for‐profit	1 [Reference]	1 [Reference]	1 [Reference]	1 [Reference]
Private	1.07 (0.85, 1.34)	1.31 (0.75, 2.29)	1.27 (0.75, 2.15)	1.16 (0.61, 2.20)
Government	1.22 (0.95, 1.56)	1.16 (0.68, 1.98)	1.38 (0.81, 2.34)	0.97 (0.54, 1.72)
Teaching status				
Nonteaching	1 [Reference]	1 [Reference]	1 [Reference]	1 [Reference]
Teaching	0.70 (0.57, 0.86)*	0.80 (0.54, 1.19)	0.73 (0.49, 1.09)	0.71 (0.46, 1.09)

a Bones and joints, prostate, and sarcoma are included in aggregate analysis but results by cancer site are not estimable. This model was adjusted for cancer site in addition to the variables shown.

* *P* < .01.

**Figure 1:**
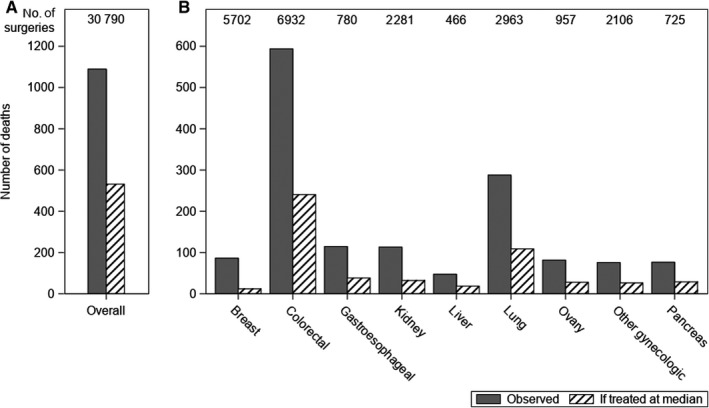
Among FFS Medicare beneficiaries undergoing cancer surgery, annual number of observed deaths and estimated deaths if poor‐performing hospitals were to improve their performance to the median hospitals’ performance. Figure a (*y*‐axis maximum of 1200) shows the results across all analyzed cancer sites; Figure b (y axis maximum of 600) for those individual cancers for which estimates were available. Findings were based on the distribution of the risk‐standardized mortality rates from the model for nonemergent surgeries adjusted for patient characteristics, overall and by cancer site. The number of lives saved was computed from the reduction in mortality if hospitals performing in the upper quartile were to improve performance to the median. The number of surgeries displayed at the top of the graph represents the number of surgeries performed at hospitals in the upper quartile. The findings overall include surgeries for bones and joints, prostate, and sarcoma, which are not estimable individually

Under a scenario in which the performance of hospitals in the upper quartile of RSMR (≥1.91%) was instead performed at a hospital with the median RSMR (1.73%), an estimated 558 lives could be saved each year among FFS Medicare beneficiaries (Figure 1, Table A2).

## 4 DISCUSSION

Quality measurement and reporting have the potential to improve outcomes through multiple mechanisms, including performance improvement within hospitals and shifting of patients to better performing ones.^33^, ^34^, ^35^ We conducted a study of 30‐day mortality following cancer surgery among FFS Medicare beneficiaries to determine if three necessary prerequisites for quality reporting are present: ease of measurement capture and reporting, statistical variation in performance, and important potential health gains through change in performance.^22^, ^23^ These prerequisites are in line with the goals of CMS’ Meaningful Measures Initiative for prioritizing areas for measurement and improvement.^20^

Our analysis provided mixed evidence regarding the promise of reporting quality measures for cancer surgery by cancer site. The outcome of 30‐day mortality can be efficiently measured at scale for cancer surgeries using FFS Medicare claims. However, for all surgeries in aggregate, 25% of hospitals performed fewer than 10 cancer surgeries and for 9 cancer sites, 25% of hospitals performed less than 3 site‐specific cancer surgery over 3 years. Low sample sizes for many hospitals pose an important problem for using quality measurement to improve cancer surgery performance at the population level. While incorporating additional years of data would increase the number of hospitals whose outcomes may be reliably reported, the tradeoff is that it reduces the timeliness of the measure. The challenge of estimating outcomes for hospitals with a low number of cases is not unique to cancer surgery but is central to measurement decisions across many health care disciplines.^32^, ^36^, ^37^, ^38^

Across all cancers in this study, as well as for most of the individual cancer sites, we observed variation between hospitals in their cancer surgical outcomes that exceeds variation due to differences in observed patient characteristics. This was supported by findings from three approaches that were mostly robust to additionally adjusting for features of the hospital and to the exclusion of emergent surgeries. Our findings are consistent with a recent study by Haneuse, et al that examined variation in 30‐day mortality following cancer surgery at 351 hospitals in California.^4^ The authors studied a younger population with a lower observed mortality rate of 0.6% (postdischarge only). But the hospital odds ratio in that study of 1.84 (95% CI 1.44, 2.34) from the model adjusted for patient and census‐based characteristics is comparable to the hospital odds ratio of 1.44 (99% CI 1.42, 1.45) we found in our model adjusted for patient characteristics. Haneuse et al examined cancer surgeries in aggregate for the primary analysis. We found that variations in individual cancer surgeries might justify reporting at the individual cancer level, beyond solely in aggregate. Our findings are also consistent with a study by Chui and colleagues that assessed the potential impact of reporting surgical mortality for lung, esophagus, gastric, and colon cancer procedures.^39^ The authors used the National Cancer Database, a clinical database that draws from hospital registry data from Commission on Cancer‐accredited facilities which are de‐identified and intended for internal quality improvement.^40^ Our study builds on this work by testing these assumptions in a dataset that identifies hospitals, which is required for public reporting. More generally, the hospital variation by cancer site we found is consistent with prior studies examining cancer‐specific mortality outcomes by hospital characteristics including volume.^1^, ^3^, ^5^, ^8^ Adjusted 30‐day mortality rates following esophagectomy for instance ranges from 20.3% at hospitals in the lowest‐volume quintile to 8.4% at hospitals in the highest‐volume quintile. Following colectomy for colon cancer, this range is 5.6%‐4.5%—a smaller absolute difference but a procedure affecting far more individuals.^5^

Last, our estimates of the magnitude of underlying variability in quality across hospitals suggest that improvements in performance could have large effects on the health of the public. We found that the difference in relative risk of the 30‐day mortality between a patient undergoing surgery at a hospital below average quality vs above average quality is at least 20% for each cancer site and exceeds 80% for breast and pancreas. If quality improvement efforts were implemented at below average hospitals or if patients were redirected for surgery at better performing hospitals, we estimate that these efforts could plausibly result in preventing more than 500 deaths among FFS Medicare beneficiaries undergoing cancer surgery each year.

Our analysis should be considered within the context of its limitations. Whether the findings would be consistent if all surgical patients, not only those with FFS Medicare coverage, were included is unknown, although there is no strong rationale why the results would not be generalizable at least directionally. The advantage of the FFS Medicare claims dataset is that it comprehensively covers the entire United States. A shortcoming is that it does not have the type of detailed information about cancer site that cancer registries contain, but prior analyses demonstrate that risk‐adjusted surgical outcome assessment is robust to the exclusion of these SEER variables.^18^, ^24^, ^31^ While we relied on the Charlson comorbidity index for our risk adjustment, which is a widely accepted method in the field, all risk adjustment can be criticized for being incomplete.^29^, ^30^, ^41^ There is controversy over the inclusion of additional information in analyses of outcome variation and in the field of quality measurement more generally.^42^, ^43^, ^44^, ^45^ We did not adjust for patient socioeconomic status or other measures of social support. We are considering additional adjustments as not relevant to this outcome given the relatively short duration of follow‐up, attributing primary responsibility to the hospital. While 30‐day mortality does not capture all aspects of surgical quality, it is an objective outcome and can be a marker of technical skill and the quality of the hospital, pre‐ and peri‐operative care, and postdischarge follow‐up. It is also used as a metric by CMS in the Hospital Compare reporting program to report performance on the quality of other types of surgery. Conducting in‐depth analyses along several dimensions of cancer surgical quality could inform future directions for hospital performance measurement.

We have found that cancer surgical mortality varies more than that which can be explained by chance or differences in treated patient populations and that collectively this variation is responsible for excess and unnecessary mortality. Quality measurement could therefore be valuable for patient decision‐making, policy evaluation, value‐based reimbursement programs, and quality improvement initiatives, with the ultimate goal of improving patient outcomes.^10^, ^46^, ^47^, ^48^, ^49^ One limitation is that quality reporting for the many hospitals in the United States that provide very low volumes of surgical cancer care will face statistical challenges. While we incorporated only data from FFS Medicare, this shortcoming could be partially ameliorated using data from more payers including commercial insurers and Medicare Advantage.

## 5 CONCLUSIONS

Our findings suggest that quality measurement and reporting of this outcome across cancers and by cancer site is worthy of serious consideration for practice and policy applications, while highlighting some of the limitations of the approach.

## CONFLICT OF INTEREST

ALS, JAL, AY, VS, and AR report no disclosures. PBB reports grants from Kaiser Permanente, grants from Laura and John Arnold Foundation, and grants from NIH Core Grant P30 CA 008748, during the conduct of the study; personal fees from American Society for Health‐System Pharmacists, personal fees from Gilead Pharmaceuticals, personal fees from WebMD, personal fees from Goldman Sachs, personal fees from Defined Health, personal fees from Vizient, personal fees from Anthem, personal fees from Excellus Health Plan, personal fees from Hematology Oncology Pharmacy Assoc, personal fees from Novartis Pharmaceuticals, personal fees from Janssen Pharmaceuticals, personal fees from Third Rock Ventures, personal fees from JMP Securities, personal fees from Genentech, personal fees from Mercer, personal fees from United Rheumatology, other from Foundation Medicine, other from Grail, personal fees from Morgan Stanley, personal fees from NYS Rheumatology Society, personal fees from Oppenheimer & Co, personal fees from Cello Health, and personal fees from Oncology Analytics, outside the submitted work. DGL reports employment at GoodRx. KSP reports stock ownership in the following companies: Johnson & Johnson, Pfizer, Catalyst Biotech, and Viking Therapeutics.

## AUTHOR CONTRIBUTIONS

Allison Lipitz‐Snyderman, Peter Bach, and Katherine Panageas were involved in study conception and design, analysis and interpretation of data, drafting of manuscript, and final approval. Jessica Lavery was involved in study conception and design, acquisition of data, analysis and interpretation of data, drafting of manuscript, critical revision of manuscript, and final approval. Diane Li, Annie Yang, Vivian Strong, and Ashley Russo were involved in analysis and interpretation of data, critical revision of manuscript, and final approval.

## Data Availability

Data sharing is not applicable to this article as no new data were created or analyzed in this study.

## Appendix A

**Table A1:** Hospital surgical volume for FFS Medicare patients: all surgeries and nonemergent surgeries only

	All surgeries			Nonemergent surgeries only		
	No. of hospitals performing surgery^a^, ^b^	Surgeries per hospital, median	IQR	No. of hospitals performing surgery^a^, ^b^	Surgeries per hospital, median	IQR
Overall	3776	34	9, 108	3753	31	8, 100
Bones and joints	302	<3	<3, 3	289	<3	<3, 3
Breast	3537	17	5, 44	3526	17	5, 44
Colorectal	3471	14	5, 34	3389	11	4, 27
Gastroesophageal	1512	<3	<3, 5	1386	<3	<3, 5
Kidney	1998	6	<3, 15	1965	6	<3, 14
Liver	556	3	<3, 7	537	3	<3, 7
Lung	1844	9	3, 23	1750	10	3, 23
Other gynecologic	1615	3	<3, 12	1563	3	<3, 12
Ovary	1137	3	<3, 10	1036	3	<3, 10
Pancreas	785	3	<3, 8	740	3	<3, 8
Prostate	1600	7	<3, 21	1594	7	<3, 21
Sarcoma	506	<3	<3, 4	464	<3	<3, 4

Note

The values that describe the interquartile range encompass hospitals with various volumes of services within those ranges. For instance, a 25th percentile value of 5 means that 25% of the hospitals performing at least one of these procedures have a total volume between 1 and 5.

The column % is shown, and the denominator is hospitals that performed one or more surgeries.

Abbreviation: FFS, fee‐for‐service; IQR: interquartile range.

a Only includes hospitals that could be matched to the American Hospital Association Survey of Hospitals 2012 (included n = 3776; total considered n = 3860 hospitals).

b Values less than three are suppressed.

**Table A2:** Calculation to determine estimated number of lives saved if poor‐performing hospitals were to improve their performance to the median hospitals' performance, overall and by cancer site

	A	B	C	D	E (=A*C)	F (=D‐E)
	Median hospital RSMR from overall model (%)	Q3 hospital RSMR from overall model (%)	Annualized no. of surgeries at hospitals ≥ Q3	Annualized observed no. of deaths at hospitals with RSMR ≥ Q3	Estimated no. of deaths if hospitals w/RSMRs ≥ Q3 improved to the median hospital RSMR	Estimated no. of lives saved
Overall	1.73	1.91	30 790	1089	531	558
Breast	0.21	0.21	5702	86	12	75
Colorectal	3.47	3.91	6932	594	241	353
Gastroesophageal	4.88	4.97	780	115	38	76
Kidney	1.41	1.43	2281	113	32	81
Liver	4.00	4.06	466	47	19	29
Lung	3.67	4.19	2963	288	109	179
Other gynecologic	1.25	1.26	2106	76	26	49
Ovary	2.93	2.98	957	82	28	54
Pancreas	3.94	3.99	725	76	29	48
